# Challenging and redefining Dollo’s law of evolution: re-appearance of lost structures

**DOI:** 10.1186/s12862-025-02405-8

**Published:** 2025-07-01

**Authors:** William R. Jackman, Vincent J. Lynch, Yann Gibert

**Affiliations:** 1 Biology Department, Bowdoin College, Brunswick, ME 04011 USA; 2 Department of Biological Sciences, University at Buffalo, SUNY, Buffalo, NY 14260 USA; 3 Department of Cell and Molecular Biology, Cancer Center and Research Institute, University of Mississippi Medical Center, Jackson, MS 39216 USA

**Keywords:** Dollo’s law, EvoDevo, Tooth, Developmental programs, Re-evolution

## Abstract

According to a modern interpretation of Dollo’s Law of irreversibility in evolution, a lost structure, is unable to reappear in evolution due to the accumulation of mutations in the genes required for its formation. However, several reports have challenged this law, even in vertebrates. Lost structures have been reported to re-appear in natural populations, as well as through spontaneous mutations, genetic engineering, and pharmacology. Some of these lost structure re-appearances are fully viable in the organism, especially while others are only detected during embryogenesis or early stages of life as the organism is unable to reach adult stages. We hypothesize that the re-appearance of lost structures can only occur if homologous structures are still fully developed in the organism, allowing for a re-utilization of the developmental pathways that are needed to re-form a lost structure. However, if no instance of the lost character remains, the re-evolution of this specific character cannot happen. Therefore, our latest results using pharmacology continue to support hat has been previously postulated: Dollo’s law of evolution remains true for lost characters that have not remained in the organism but should be amended for the re-apparition of lost structures with serial homologous characters present in any form in the organism.

## Introduction

Over 130 years ago, Belgian-French paleontologist Louis Dollo stated that “an organism never returns exactly to a former state, even if it finds itself placed in conditions of existence identical to those in which it has previously lived” [1]. Based on this statement, Simpson [2] formulated a simple evolutionary concept which is known now as Dollo’s law of irreversibility, stating that evolution is not reversible and that lost structures cannot re-appear. However, there is more complexity to this statement than was perhaps originally thought. Several evolutionary biologists, including Richard Dawkins, have highlighted the statistical improbability for a lost tissue/structure to follow the exact same genetics, genomics, physiological, cellular and molecular pathways or trajectories twice in either direction (evolution or devolution) [3]. For the first part of this review we will focus on the commonly accepted Dollo’s law of irreversibility during evolution, and in a later part put our new perspectives on Dollo’s law in combination with the latest understanding from examples of apparent evolutionary reversals.

## Example of Dollo’s law of irreversibility

Numerous examples come to mind to validate Dollo’s law of irreversibility: the loss of teeth in modern birds, the loss of limbs in snakes, the loss of hindlimbs in Cetaceans and hair in most Cetaceans species, and the loss of oral teeth in Cypriniforms, to list a few. The loss of each of these structures has an evolutionary explanation, such as evolving leglessness to help in the locomotion of crawling animals such as snakes or swimming animals such as dolphins and whales. In a somewhat more complex example, the loss of teeth in modern birds has been argued to have been a secondary effect of selection for the beak [4]. Yang and Sander argue that tooth formation was likely a rate-limiting factor for embryonic developmental speed and that tooth loss was a side-effect of selection for fast developing embryos [4]. Regardless of the exact reasons for their loss, none of these complex structures have re-appeared during evolution, indicating that the Dollo’s law may have some truth based on paleontological and observational analysis. However, most rules are made to be broken, and numerous violations of the Dollo’s law have been reported in the literature, prompting for a re-evaluation or at least amending of this law. Some widely presented examples of such apparent violation of Dollo’s law come for lizards where lost toes and oviparity were able to re-evolved after having been once lost in these species [5, 6] or upper body muscle reversions in primates [7]. Collin and Miglietta [8], used the latest advances in phylogenetic methodologies to challenge Dollo’s law. The authors argue that it is easily conceivable that gene silencing from mutations in regulatory sequence, which does not require the deletion of the gene or deleterious mutations accumulation in the gene [9, 10], can happen in a gene with a single function: i.e. changes in the control circuit of a gene with a specific function of the lens [11], enamalin or hemoglobin. However, if a gene is silenced in the long-term, due to the lack of selection pressure, mutations will inevitably accumulate in its coding sequence. Marshall et al., estimated that the maximum time period over which a silenced gene could be re-activated with efficacy is no longer that 6 million years [9]. In other words, the encoded function of an unused protein-coding gene could not be retained for more than 6 myr due to the accumulation of random mutations. Based on the rate of gene loss in duplicated genes after whole genome duplication, Colin and Miglietta proposed a longer estimation and came to the conclusion that unused genes will lose their ability to be reactivated for 8 myr and would certainly not last longer than 16–24 myr [8]. This scenario of random mutation accumulating in unused genes is only valid for a minority of genes as it only applies to genes with a unique function (i.e. not pleiotropic). As most genes in vertebrate genomes have several different effects, they are therefore expected to maintain their expression in other cells/tissues even when a specific character is lost. For example, the transcription factor *dlx2* is expressed in several independent tissues in mammals such as the auditory system, the branchial arches, the nervous system, and the respiratory system [12, 13]. Therefore, if one specific character requiring the expression of *dlx2* is lost in a particular taxon during evolution, selection pressure will still be active on *dlx2* as its expression/function will still be needed for the other characters in which it is implicated, preventing the accumulation of random genetic mutation in this protein-coding gene. Therefore, the pleiotropic functions of genes will maintain their sequence integrity and allow for re-activation of a lost function or a lost character, as long as the genetic information needed for regulation of expression of a particular gene remains available. Furthermore, if a gene is implicated in any type of mandatory developmental pathway, there will be strong selection pressure to maintain the function of this gene even if larval or adult structure implicating this particular gene is lost. Therefore, developmental pathway retention could be, as pleiotropic functions, a strong reason why the genetic information may remain unaffected in a gene even when the complex trait expressing this particular gene has been lost. Another important factor to keep in mind when dealing with sequence integrity of genes originally present in a lost structure is their genetic architecture: the underlying genetic basis of phenotypic trait and its variational properties [14]. As Collin and Miglietta proposed: “large genomics databases and evo-devo studies are showing how the underlying developmental pathways and genetic architecture can be retained after the loss of a character” [8], however complete loss of either of them: developmental pathway and/or genetic architecture will prevent the reappearance of a lost complex trait.

## Phylogenetic method and Dollo’s law violation

Phylogenetic comparative methods are most often used to demonstrate that lost complex traits have re-evolved. Kurtén [15], for example, used an ad hoc parsimony argument to justify that the molar M_2_ re-evolved in Eurasian lynx because all other extant felids lack M_2_; thus, because the Eurasian lynx is relatively deeply nested in felids, and the presence of M_2_ is polymorphic in 3.4−27% of Eurasian lynx populations, it must have recently re-emerged. Subsequently, parsimony [16, 17], maximum likelihood [18–20], and Bayesian methods [21] have been developed that can be used to explicitly test whether there is statistically significant evidence that lost complex traits have re-evolved; these methods usually test at least two models, one in which reversals are not allowed versus models in which reversals are allowed if the “best” model allows for reversals re-evolution is supported. Phylogenetic tests of Dollo’s law, however, can be misled by several processes and should be used with caution. One limitation is that it is based on the assumption that the phylogeny is correct, meaning that an inaccurate phylogeny (regardless of the method used: NJ, MP or ML) will induce a strong bias in the subsequence analysis [8]. Another example is that maximum likelihood and Bayesian methods often assign the probability of the root state to be equal between the different character states or the equilibrium frequency of the observed character states; both of these assumptions are invalid under a Dollo model [22]. A character’s state may also affect lineage speciation and extinction rates, which biases ancestral state reconstruction [22] and leads to erroneous rejections of a character’s irreversibility. Other processes that may mislead phylogenetic inferences of re-evolution include incomplete lineage sorting, hybridization [23], and phylogenetic error but these processes can be accommodated with more complex models of character evolution [24]. These limitations of phylogenetic tests of complex trait irreversibly indicate that inferences of re-evolution of lost complex characters should be supported by additional kinds of information, in particular, anatomical, physiological, and developmental data [25, 26].

## Dollo’s law violation during tooth formation

Numerous examples of alleged Dollo’s law violation exist in the literature. Re-evolution of complex characters has been suggested in numerous vertebrates and for several tissues/organs including digits in lizards [27], re-evolution of an aquatic larval stage in salamanders [28], thigh muscles in the common Myna and the Fox Sparrow [29], or even the re-evolution of the adaptive change from blue to red flowers in the morning glory cypress wine [30] just to name a few. However, perhaps most of examples of the re-evolution of complex traits in vertebrates concern teeth. Tooth re-evolution is not as rare as hen’s teeth, in fact one of the most famous examples of tooth re-evolution was described in 2006 where Harris and colleagues [31] used the naturally occurring *talpid*^*2*^ chicken mutant (an autosomal recessive mutation affecting several systems leading to polydactyly and oral-facial malformation [32]) to identify partial tooth formation (these newly formed teeth lack enamel) on the mandibula and the heavily deformed maxilla. The avian *talpid*^*2*^ mutant is due to a deletion mutation in the *C2CD3* gene, centriolar protein that is required for ciliogenesis [33]. The *talpid*^*2*^ mutant is lethal before hatching, therefore no adult chickens were ever observed bearing teeth (or partially formed teeth). This limitation to early stages of development is not the case for tooth re-evolution in other vertebrate species. One of the first examples of tooth re-evolution comes from a time-calibrated phylogeny analysis of 170 amphibian species. In his analysis, Wiens showed that the mandibular teeth were lost in the ancestor of modern frogs at least 230 myr ago and have been re-gained in the last 5 to 17 myr [34]. This particular frog species, *Gastrotheca guentheri* (*G. guentheri*) or Guenther’s marsupial frog is famous for being the only known frog species with genuine teeth in the lower jaw, while all other frog species can only bear teeth on the upper jaw and palate [35]. Unlike the chicken example described above, *G. guentheri* are viable as adults and perfectly adapted to their natural environment, making this frog species an apparent true violator of Dollo’s law. One caveat in the time frame proposed by Wiems regarding the loss and reappearance of mandibular teeth are, as acknowledged by the author himself, the assumptions that were made: in his essay, Wiens [34] stated that the following assumptions could be challenged: he assumed that 1- the original authors trait reconstruction are correct; 2- the estimated divergence times are correct and 3- it is unknown where on the phylogenetic tree a branch a state occurs. These assumptions, even if valid only to a certain extent, may induce a strong bias in the conclusions reached regarding the time elapsed between the loss of a complex trait and its reappearance [34]. The stretch of tooth re-evolution does not stop in amphibians as at least one mammalian species, the Eurasian lynx (*Lynx lynx*) has been documented for the re-appearance of the molar M_2_ that is absent in all other modern felids but was present in ancestral cats [15].

## A simple developmental model of tooth re-evolution in vertebrates?

**Fig. 1:**
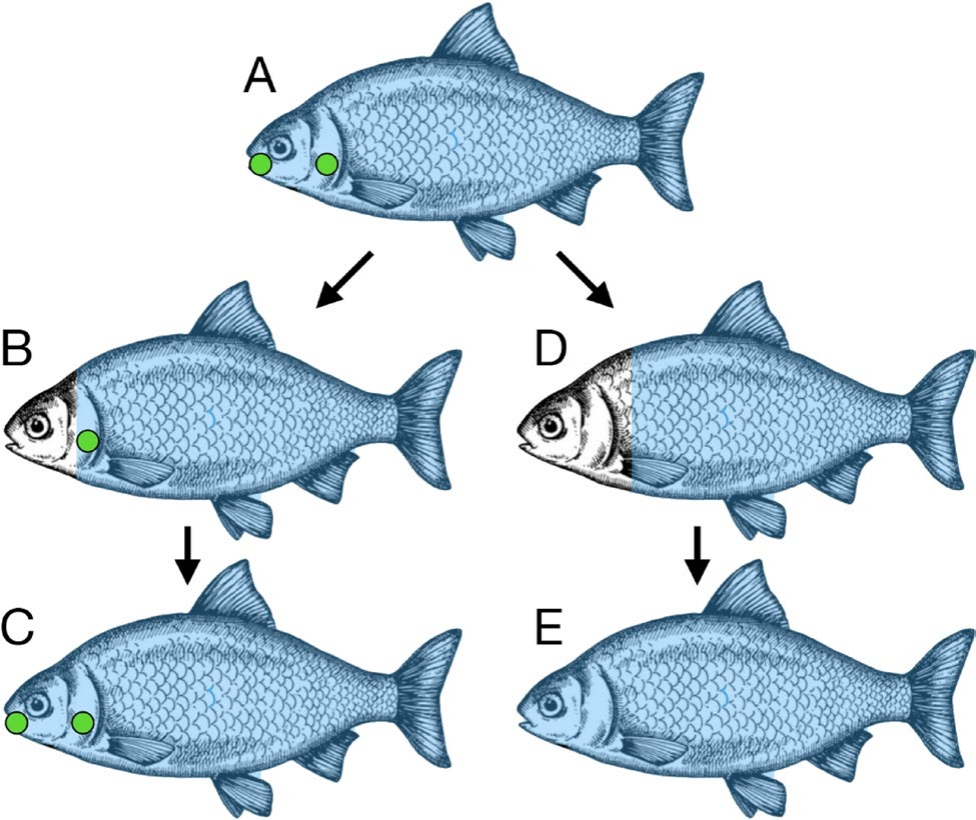
Model of character loss and reappearance. **A** An ancestral species with developmental positional information (blue, e.g. RA inducing Hox expression) that allows for the developmental program for character formation (green, e.g. Shh inducing tooth-specific protein expression in oral and pharyngeal teeth) to form in multiple locations. **B** A shift in the positional information eliminates some but not all of the occurrences of the character (e.g. elimination of oral teeth but not pharyngeal teeth), thus the developmental program is preserved, allowing a later return of the positional signal **C** to re-introduce the lost character (e.g. oral teeth). However, if the positional information shifts such that it removes the character completely (e.g. no teeth at all) (**D**), the developmental program will be lost via mutation, and even a later return of the positional signal (**E**) won’t be able to recover the lost character

To go back to the previous example of the *dlx2* gene pleiotropic effects (see paragraph 2), we previously demonstrated, by using a cis-regulatory enhancer of *dlx2b* of zebrafish, a species devoid of oral teeth, that this enhancer is able to drive the expression of a reporter gene in oral teeth in a fish species that normally develops oral teeth: the Mexican tetra [36]. The *dlx2b* scenario in fish implies that even though a specific character, in this case oral teeth, has been lost in zebrafish, the zebrafish *dlx2b* enhancer has retained the ability to drive expression in this lost character when incorporated in a related species (the Mexican tetra) that has retained this specific character. Unlike what was proposed as a time frame before all information is lost for a gene with unique function: 6 to 24 myr depending on the model used, it was estimated that the information retention in the cis-regulatory element of the *dlx2b* genes has survived for around 50 myr [36]. Moreover, recently our laboratory studied the re-evolution of oral teeth in fish. The Cypriniforms lineage, which includes the popular zebrafish laboratory species, lost their oral teeth around 50 to 100 Mya [37] and have solely maintained teeth in the ventral posterior pharynx on the last ceratobranchial arch [38]. We previously demonstrated that retinoic acid (RA) cell signaling is necessary for the induction of pharyngeal teeth in zebrafish [39] and that experimentally applied excess RA was sufficient to induce the formation of supernumerary pharyngeal teeth in the anterior pharynx [40]. Additionally, pharmacologically manipulating RA signaling by blocking the actions of the endogenous enzymes that degrade RA using Talarozole, we were able to induce the formation of tooth germs with partially calcified teeth in the oral cavity of the zebrafish embryo [41]. Therefore, do our experiments in zebrafish conflict with Dollo’s law of evolution, as we were able to initiate the formation of structures that have been lost for over at least 50 Mya? Is the re-evolution of lost structures due to genetic mutations/manipulations or the use of pharmacology as valid as a naturally occurring re-emergence of a lost structure? The main differences with the re-appearance of oral teeth germs in zebrafish and the formation of a lost molar M_2_ in lynx is that in the case of the lynx, the re-emerged structure is fully functional in adults and is observed in the wild without any external input, while for oral teeth in zebrafish an experimenter was required to “boost” the level of endogenous RA in the oral region to allow for teeth to be induced. Nevertheless, both re-appearances, regardless of whether they are natural or pharmacologically induced, seem to conflict with Dollo’s law of evolution. Lost teeth have been documented to re-appear in several vertebrate groups: fish (zebrafish), amphibians (Guenther’s marsupial frog), and mammals (lynx), but in all of these species, teeth are present elsewhere in the body plan, meaning that the genetics, developmental programs, cellular biology, and molecular aspects of tooth formation are present and fully functional in these organisms. A striking example to confirm the importance of retaining the genetic information to form a specific structure comes from birds. Comparison of genomic data in tetrapods focusing on the secretory calcium-binding phosphoproteins (SCPP) genes, involved in tissue mineralization, revealed that the enamel SCPP degenerated in the bird lineage after these animals lost their teeth during the Late Cretaceous [42]. The loss of this specific tissue mineralization gene makes the formation of functional teeth with enamel impossible in birds. Therefore, one might argue that the re-appearance of lost traits is not a true re-appearance as the biological programs required for their development was always present in the organism and never lost, but singly re-used in a different context or location for the formation of a lost structure. In other words, we propose that as long as an organism retains all the genetic information required for the formation of a specific character, such as a tooth, then the developmental program required for tooth formation is not lost and under specific conditions (naturally or induced by the experimenter) the genetic and cellular programs required for the initiation of serial homologous characters can be re-initiated, reviving the formation of structures that were once lost. Therefore, is our study of reappearance of oral tooth germs in zebrafish just another example challenging Dollo’s law of evolution? Well not exactly. Our study is different from what has been reported to date in two ways: first we used pharmacology to prevent the degradation of a specific pathway: the retinoic acid pathway in a region that is normally devoid of RA signaling: the oral cavity in zebrafish. Second, blocking RA degradation is enough to activate the formation of dorsal oral tooth germs in zebrafish [41]. This is important because on the top of not having oral teeth, zebrafish are also normally devoid of dorsal teeth. Therefore, if the hypothesis we are trying to examine is that retaining a homologous structure is vital for the reappearance of lost structures even a at different location (ventral pharynx versus oral cavity)? How can this be valid in a species that did not retain any dorsal tooth at all? To answer this question, we first have to consider the following question: are dorsal teeth homologous to ventral teeth? The definition of homology which was coined by Richard Owen in 1843 defines it as pre-evolutionary, nontransformative and applied to fully formed structures in animals [43]. The definition of homology given by Owen, imply that to be considered homologous, two characters that might seems different must be classified as “same” (in the sense of sameness or similar). Can upper and lower teeth in fish be classified as homologous in structure but differ only by their localization? Minelli argues a need for a clear-cut distinction between positional and special homology (special homology is based on the identity of features of a body structure while positional homology refers to the location of a structure in the body) and even proposed to abandon the concept of homology as an all-or-nothing relation [44]. However, in his essay, Brian Hall proposed a more modern definition of homology by defining it as the presence of the same character in two lineages that share a common ancestor [45]. Therefore, based on the definition given by Hall, it seems that upper and lower teeth in fish fit the criteria for homologous structures. However, in practicality it might be highly challenging to fully resolve homology especially if a specific character does not have any homologous suture that remained in the organism. So how can we address the question of upper versus lower teeth homology in fish? As often the answer came by the mean of experimentation: overexpression of the Ectodysplasin (Eda) gene in zebrafish is able to restore the formation of upper teeth in the posterior pharynx, but not in the oral cavity [46]. Therefore, restoring a signal, Eda in this case, in a zebrafish region where it has been lost through evolution is enough to re-activate the formation of a lost structures such as the upper teeth in zebrafish. In the case of Eda overexpression, the authors failed to identify oral tooth germs and conclude that multiple genetic changes would be required to restore oral teeth formation in zebrafish [46]. However, our recent work showed that it is not the case. We argue that RA signaling works upstream in the “tooth formation cascade” meaning that restoring an early instructive and inductive signal like RA signaling in the oral cavity can in turn activate one or several downstream signaling pathways that will work together to establish the formation of oral tooth germs. Although the homology between upper and lower teeth has not yet fully established in fish, our data and previously published data show that they share a common ancestor and therefore can be consider homologous. Even though we did not provide definitive evidence about their homology (and one might never be able to as it will be highly challenging to demonstrate this), the fact of the matter is that upper teeth can be re-activated and develop in zebrafish after pharmacological supplement of the missing signaling molecule. We therefore propose that Dollo’s law of evolution still holds true for unique character not present anymore in the organism, but that the idea needs to be amended when dealing with the re-appearance of an organ or tissue when serial homologous characters or characters sharing a same common ancestor still remain (Fig. 1). We also propose that this amendment of Dollo’s law can be extended beyond the specific example of lost tooth re-appearance. By looking at the earlier examples cited in this paper, one will realize that the re-appearance of lost structures all fall under the category of still-existing homologous characters in the organism: digits in lizards, muscles in foxes, and the re-evolution of already existing larval stages in salamanders. One common trait shared by the examples cited in the previous sentence is that they are all related to meristic characters: a complex trait that can be used to describe a particular species, or used to identify unknown species. Several authors have suggested that serially repeated structures constitute a special case in regards to Dollo’s law of irreversibility, because the genetic and architecture to develop these serial structures continue to be present and active in the organism when their number of this structure decreases: For example, Willi Hennig in his published work on theoretical taxonomy in 1950, translated into English in 1966 used modern phylogeny analysis to propose this novel hypothesis [47]. Over 50 years ago, Stephen J Gould hypothesized that gains in number of meristic traits could be classified as Dollo’s law violation [48]. More recently Marshall et al., proposed that the re-apparition of the Molar M2 in lynx represents a gain of a lost structure after a long period of time may not represent re-activation of any silenced genes but rather represents changes in the level of gene activity controlling the organization of the molarization field in this species [9].

Building on this hypothesis, Galis and collaborators presented data on the irreversibility of digit loss in the lizard genus *Bachia* and also on teeth using these meristic traits to challenge the irreversibility of lost character depicted in Dollo’s Law [5]. We also proposed that reappearance of meristic trait such as tooth in zebrafish [41], challenges what is commonly accepted in Dollo’s law. We can push further this reasoning by concluding that loss of any meristic character could be classified as a non-violation to Dollo’s law per se when re-gained as meristic characters fall in a specific and unique type of lost trait. However, even when in repeated structures, the genetic and developmental information is retained, still re-evolution appears to be impossible. A good example of this is the loss of one pair of limbs in marine mammals. However, in that case once might argue the hind and for limb, although both limbs do not share similar developmental pathway as they use a different T-box gene as initiating transcription factor (*tbx5* for the fore limb and *tbx4* for the hindlimb [49]).

This notion that serial repeated element may fall outside of what the Dollo’s law englobes was already suggested by Wake et al., in 2011 who agued that “serial repeated structures (like teeth) have re-evolved but the developmental genetic and morphogenetic underpinnings are likely to have been retained” [50]. The authors also stated that serial homologous structure can re-evolved in different position within animal bodies [50]. Although this latter statement is true the position in which a lost complex trait re-evolve is near within the same organ/tissue in which it has been retained (eg. upper versus lower jaw). However, our recently published story also brings novel notions to this debate: the re-activation of complex trait lost in a different location: upper oral teeth in zebrafish which is devoid of both oral and upper teeth and re-activation of a master signaling cascade such as RA is sufficient to re-activate downstream signaling cascades to re-initiate oral tooth germs induction in zebrafish.

## Loophole in our simple model?

Based on the examples presented in the previous section, our proposed amendment of Dollo’s law of evolution holds true. However, earlier we mentioned the re-appearance of teeth in a particular chicken genotype [31]. Is the talpid^2^ chicken example an exception to this newly proposed amendment of Dollo’s law, as chickens are devoid of any king of tooth in their beak or anywhere else in their body? Although modern birds do not develop teeth, they all develop a specialized keratinized structure, the rhamphotheca, that might still retain most of the genetic information to form a tooth. Furthermore, the talpid^2^ chickens have teeth with pre-dentin matrix but not true dentin or enamel. Thus, they partly regain the structure, but it is still not complete, therefore this doesn’t really invalidate the ‘amendment’ to Dollo’s law. One might argue that chicken and bird in general also possess a specialized structure called egg tooth. However, in birds, the egg tooth is formed from a thickening of epidermis at the tip of the snout and besides being called an egg tooth, this structure is not homologous to a true tooth and cannot be accounted as a tooth formation. Therefore because of the formation of an incomplete and non-functional tooth and the presence of the rhamphotheca, we believe that our addition to the current Dollo’s law of evolution still holds true. Other published examples support our hypothesis we are making in this paper: the article “Are there general laws for digit evolution in squamates? The loss and re-evolution of digits in a clade of fossorial lizards (*Brachymeles*,* Scincinae*)” published in 2018 [51] supports our hypothesis that the re-evolution of complex traits may be more likely if a serial homolog remains (digits, not limbs). However, the reversion to oviparity in reptiles is contested, even in the case of Eryx, because it is not clear these are true reversions rather than the evolution of an analogous (i.e., not homologous) egg-shell like structure [52]. This current essay is focused on the gain/loss of complex traits, rather than traits regulated by a single gene, the example of shell coiling is a very good one because it too suggests that a complex trait can re-evolve if there is some remnant of the developmental genetic program for that trait [53, 54], as is the loss/gain of sex combs: a recently evolved, male-specific morphological structure composed of modified bristles in *Drosophila bipectinate* [55]. Sex combs were lost in the ananassae species subgroup (fruit flies species) and subsequently re-evolved, around 12 million years later, in *D. bipectinata* and its sibling species [55]. In their recently published review, Elmer and Clobert (2025) present different examples of Dollo’s law of irreversibility in the post-genomic age [56].

## Conclusions

In this review, we have tried to convince the reader that Dollo’s law of evolution as currently stated does not hold true for the reappearance of lost characters for which at least a single homologous character is still normally present (e.g. a tooth). Because this remaining character is normally formed, this logically implies that all the genetic, developmental, cellular and molecular cues are fully functional so that proper development of this character can occur. This means that the selection pressure is actively acting of the developmental pathways to maintain the formation of this character and therefore with the right natural mutations or biological modifications inflicted by the experimenter, these fully functional and evolutionary selected pathways can be re-activated by cells in part of the body where they have not been active in million years of evolution, thus, re-initiating the formation of a character that was lost. However, if the formation a at least a single character has not been under selection pressure by evolution, the re-evolution of this specific character will be lost due to accumulations of mutations in genes and signaling pathway required for its development. By pushing forward this theory a bit more, one could consider the re-initiation of developmental pathways in adults. For example, normal development in vertebrates leads to the formation of two forelimbs and two hindlimbs, and we know several vertebrate species such as zebrafish and newts that can fully regenerates their limbs, while this capacity has been lost in mammals. But if we are following our logic from the amendment of Dollo’s law of evolution, the genes and signaling pathway to re-form a limb once it has been lost in humans are still present in the genome but in a silent stage that we are unable to re-initiate. However, unlike what was generally accepted, limb regeneration is not solely due to a re-activation of developmental processes to form *de novo* a new limb. A recent study in newt demonstrated that a *fgf10* mutant that is unable to form hindlimb during development, due to the fundamental role of *fgf10* during hindlimb formation, can regenerate normal hindlimb despite lacking this functional hindlimb master gene required during embryogenesis [57]. Therefore, the genetic tools and pathways used for the *de novo* formation of a limb are still under some selection pressure even if they are not part of the original developmental processes. Studies challenging the Dollo’s law of evolution may teach us that if we find the cues to properly re-initiated these genes and pathways, then the re-appearance of a lost limb might not be impossible. Only future studies will tell.

## Data Availability

No datasets were generated or analysed during the current study.

## Abbreviations

GFP: Green fluorescent protein
hpf: Hours post fertilization
M: Molar
ML: Maximum likelihood
MP: Maximum parsimony
Myr: Million years
NJ: Neighbor joining
RA: Retinoic acid
